# Cognitive Health and Quality of Life After Surviving Sepsis: A Narrative Review

**DOI:** 10.1177/08850666251340631

**Published:** 2025-05-16

**Authors:** Khalia Ackermann, Nanda Aryal, Johanna Westbrook, Ling Li

**Affiliations:** 1 Centre for Health Systems and Safety Research, Australian Institute of Health Innovation, 7788 Macquarie University, Sydney, Australia

**Keywords:** sepsis, cognitive dysfunction, mental health, quality of life, functional status

## Abstract

**Purpose of the research:**

Sepsis is a leading cause of disease and affects approximately a third of ICU patients worldwide. Despite the rising number of sepsis survivors, the burden of cognitive and quality of life related post-sepsis morbidities remains understudied. This narrative review aimed to summarize and discuss current research investigating the quality of life and the burden of cognitive, mental, and functional health morbidities in sepsis survivors at different stages of life.

**Major findings:**

Sepsis survivors of all ages were affected by cognitive dysfunction, with very preterm neonatal sepsis survivors reporting higher odds of neurodevelopmental disabilities, childhood sepsis survivors reporting delayed development, and adult sepsis survivors reporting cognitive decline, including a higher risk of dementia. Mental health concerns were reported in both survivors and family members, with limited mixed evidence for post-traumatic stress disorder, depression, suicide, and anxiety. Survivor functional status is frequently impacted in diverse ways, with both physical and mental changes often inhibiting daily life. Lastly, the impact of sepsis on survivor quality of life is mixed. While sepsis survivors frequently report poorer quality of life compared to the general population, studies have reported no difference in quality of life when comparing sepsis survivors with other critical illness survivors.

**Conclusions:**

Sepsis impacts the quality of life and cognitive, mental, and functional health in numerous diverse ways across the lifespan. Future research should focus on sepsis survivorship in children, and the mental health burden of sepsis across all age groups.

## Introduction

Sepsis, a condition characterised by life-threatening organ dysfunction in the presence of suspected infection, is a leading cause of disease worldwide.^1^ An estimated 48.9 million people across the globe had sepsis in 2017, of which 20.3 million were children under the age of five.^2^ Further to this, there were 11 million sepsis-associated deaths reported in 2017.^2^ The sepsis survival rate is improving with the number of sepsis-related deaths reported to have decreased by 29.7% (from 15.7 to 11.0 million) during 1990–2017.^2^ Consequently, there are more sepsis survivors now than ever before.

Following discharge from the initial sepsis hospitalisation, survivors often suffer long lasting physical, cognitive, emotional, and social impacts.^3-5^ These ongoing effects are also known as ‘Post Sepsis Syndrome’ (PSS).^3^ PSS presentation is diverse, and many survivors report high rates of morbidities involving cognitive health, quality of life, mental health concerns, and functional limitations.^3,4^ A recent qualitative study by Skov et al,^6^ reported that sepsis survivors found psychological and cognitive impairments to be influential concerns in their daily lives.

While evidence on the burden of sepsis survivorship is recent and growing, it remains a critical area of study given the rising number of sepsis survivors.^3,4,7^ Quality of life and cognitive, mental, and functional health concerns have been established in critical and intensive care survivors.^8-12^ As an estimated third of critical care patients have sepsis,^13^ understanding the burden of these health concerns in sepsis survivors specifically may be valuable. Preliminary searches of the literature demonstrated emerging research with extremely diverse outcomes, populations, study designs, and distinct subtopics. Narrative reviews allow for a broad overview of these topics while still reflecting the diversity of studies.^14^ An important gap in current narrative reviews is a failure to investigate patients across different life stages.^3,4,15-19^ Children and adults can have different sepsis presentations and may subsequently display different post-sepsis sequalae.^1,20^ Therefore, this narrative review aimed to summarize and discuss the research investigating the quality of life and burden of cognitive, mental, and functional health morbidities in sepsis survivors at different stages of life.

## Method

**Figure 1.**
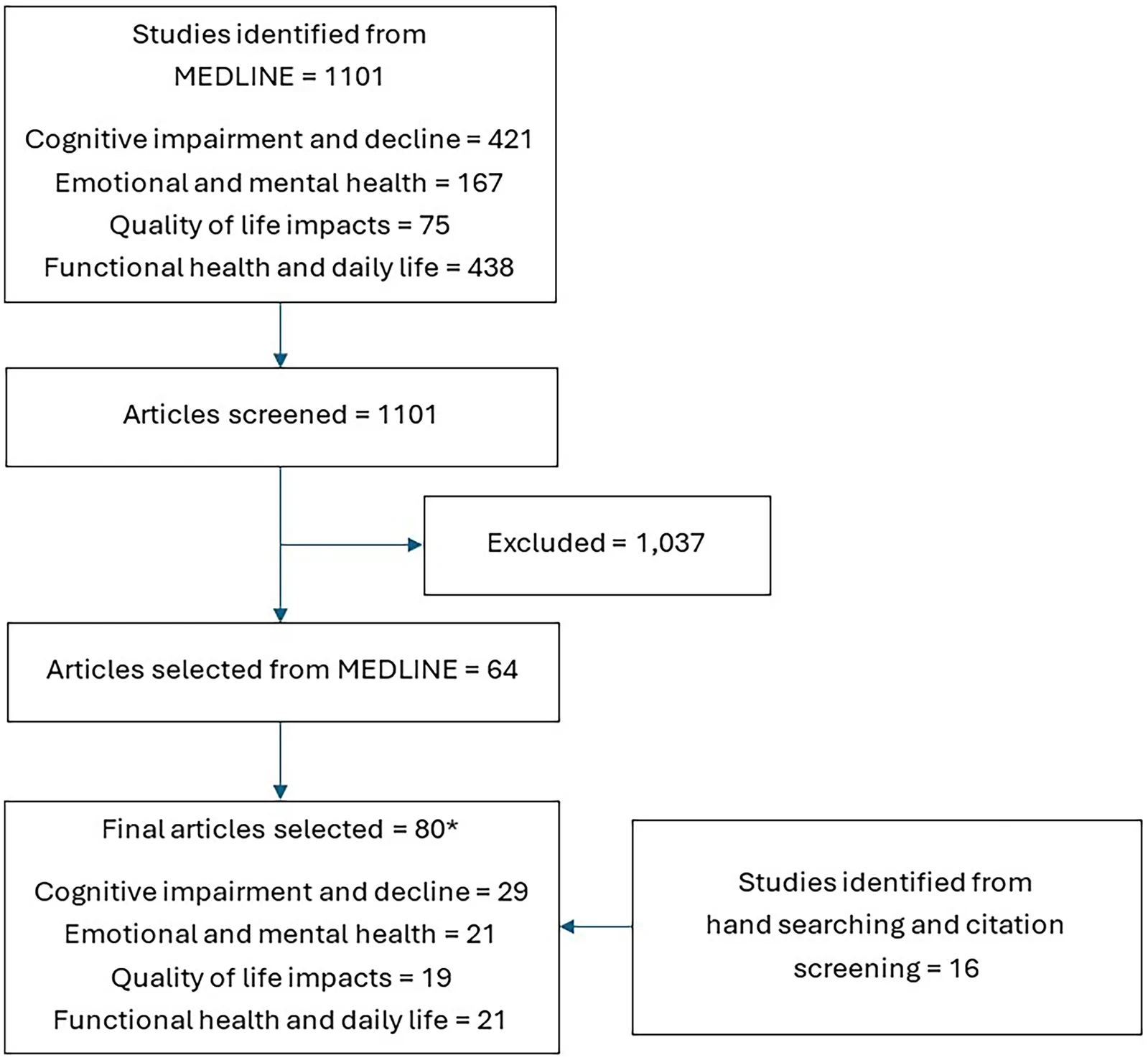
Flow Diagram of Selected Studies. *Some Studies Were Selected for Inclusion in More Than one Section and Therefore the Section Totals do not add up to the Total Studies Selected.

Search strategies geared towards cognitive decline, mental health, quality of life, and functional status outcomes in sepsis survivors (Appendix A) were used to search MEDLINE in March 2025. Further studies were retrieved through hand-searching and the reference lists of relevant review articles. Titles, abstracts, and full texts of returned search results were screened and a selection of relevant studies that introduced unique themes or results related to the review aim were included (Figure 1). When a systematic review was available on a specific topic, it was selected in place of original studies. Study results were characterised iteratively into four sections as per the study aims: (i) new cognitive impairment and/or decline, (ii) mental and emotional health, (iii) changes in functional status and daily life, and (iv) quality of life impacts.

Included studies had to investigate the quality of life or any aspect of cognitive, mental, and functional health in human sepsis survivors. Only studies published in english and reporting original research were included. For this review, we considered studies investigating any aspect of patient health related to the brain, including cognitive changes, brain damage or impairments, and psychological or emotional health impacts to be relevant to cognitive and mental health respectively. Any aspect of patient health relating to everyday function or changes in activities of daily living and independence status were considered relevant to functional health. Finally, studies investigating quality of life were included if they used either a validated quality of life instrument or qualitatively investigated patients’ perspectives on their post sepsis experiences. The literature on sepsis sequalae is extremely broad.^3,4^ Many sequalae, such as the four domains targeted by this review, have diverse literature and definitions. To allow for adequate breadth of coverage of these domains across different life stages we considered more well-characterized sepsis sequalae such as mortality, readmissions, and non-cognitive chronic diseases out of scope of this review. Therefore, studies only investigating these outcomes were excluded. The scale for the quality assessment of narrative review articles (SANRA) was used as a guide during manuscript preparation.^21^

## Results

**Figure 2.**
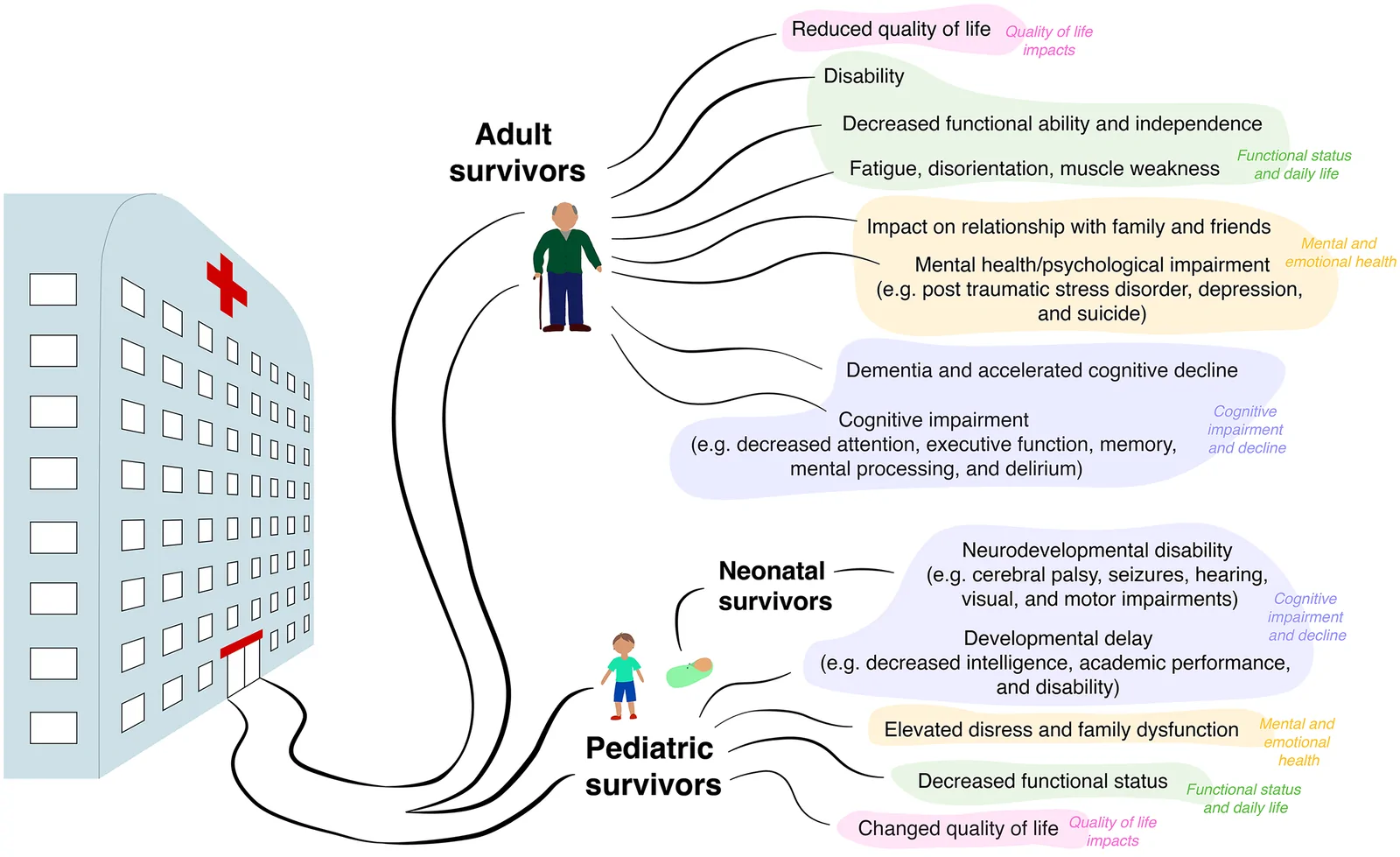
Summary of the Potential Impact of Sepsis on Quality of Life and Cognitive, Mental, and Functional Health Across life Stages.

Figure 2 summarises the main quality of life and cognitive, mental, and functional health sequalae currently reported to impact sepsis survivors at each stage of life. All studies selected for data extraction are listed in Appendix B.

### New Cognitive Impairment and/or Decline

#### Pediatric Sepsis Survivors

##### Newborn Children

Infants and young children are particularly vulnerable to sepsis, making up a large proportion of the disease burden.^2,22^ In 2019, 6.31 million newborns had an incident case of neonatal sepsis globally, with approximately 230 000 sepsis-associated deaths.^23^ There is an established association between neurodevelopmental outcomes in very pre-term and very low birth weight infants and neonatal sepsis, as reported in three systematic reviews and meta-analyses by Cai et al,^24^ Alshaikh et al,^25^ and Bakhuizen et al.^26^

The most recent of these reviews by Cai et al^24^ included 24 studies, 14 of which were analyzed by meta-analysis. The findings suggest the odds of very preterm and very low birth weight newborns with sepsis developing neurodevelopmental disabilities are 3.18 (95% CI 2.29-4.41) times higher than those without sepsis.^24^ This association persists for more than 36 months after sepsis admission, as shown by a sub-analysis of four studies.^24^ Meta-analyses performed by Alshaikh et al^25^ and Bakhuizen et al,^26^ and a 2021 narrative review,^19^ reported neonatal sepsis was associated with the development of neurodevelopmental, motor-skill, visual, hearing and cognitive impairments, as well as cerebral palsy, seizures, and oxygen requirements. Kurul et al^27^ reported the risk of neurodevelopmental and motor impairments was higher in infants with increased C-reactive protein levels (a marker of inflammation) and sepsis severity as measured by the neonatal sequential organ failure assessment. Furthermore, newborns who had sepsis more than once, also known as recurrent sepsis, had a higher risk of poor motor development compared to newborns with a single sepsis incident.^27,28^

##### Non-Infant Children

Sepsis impacts the cognition and neurodevelopment of children, not just as a newborn, but at all ages and stages of development.^29-32^ A large cohort study by Savioli et al^31^ found 50.5% of children who survived a ‘confirmed’ sepsis incident were developmentally delayed by age five years compared to 28.2% of children who survived a ‘suspected’ sepsis incident, and 22.2% of children who did not have sepsis. Surviving pediatric sepsis has also been associated with lower intelligence, verbal recall, and higher neuropsychologic impairment three to six months post-discharge compared to healthy controls.^29^ The same study also reported a deterioration in academic performance in pediatric sepsis survivors, characterised by difficulties keeping up with schoolwork, dividing attention, or considering multiple possible solutions.^29^ In addition, children with septic-associated encephalopathy (SAE), a type of brain dysfunction due to sepsis, have poorer performance in intelligence and general development tests three to nine months post-discharge.^30^

A 2021 study set in an Indian pediatric intensive care unit (PICU)^32^ found that while 28.1% of childhood sepsis survivors had a new cognitive disability post-PICU discharge, only 14% still had this new disability after one year. In contrast, it is often hypothesised that children may “grow into deficits”, suggesting brain damage occurring early in life can cumulatively affect development, leading any negative impacts to become more apparent with increasing age.^33^

#### Adult Sepsis Survivors

Adult sepsis survivors report short, medium, and long-term cognitive impairments.^34^ A landmark study by Iwashyna et al^35^ investigating adults 50 years and older reported the odds of severe sepsis survivors having moderate to severe cognitive impairment were 3.34 times higher than before sepsis. A German cohort study, published in 2021, reported 18.5% of at-risk sepsis survivors had a new cognitive diagnosis using retrospective claims data.^36^ A second, prospective, German cohort study published in 2024 found that 57.9% of intensive care unit (ICU) sepsis survivors self-reported new onset of a cognitive impairment.^37^ In addition, sepsis patients experience faster cognitive decline over time compared to their pre-sepsis trajectory and to patients without sepsis.^38^ Survivors with ongoing cognitive impairments also demonstrated accelerated brain aging.^39^

Adult sepsis patients are commonly afflicted by septic-associated encephalopathy (SAE).^17,40^ The presentation of SAE can range from delirium to coma and may occur in up to 70% of sepsis patients, though the reported incidence rates are diverse.^40^ SAE is associated with higher mortality and poor long-term cognitive outcomes, and can affect attention, verbal fluency, executive function, memory, and mental processing speed.^40^ In particular, delirium associated with sepsis and SAE correlates consistently with the development of long-term cognitive impairments,^17,41^ with the length a patient is delirious associated with poorer outcomes.^41^

In older sepsis survivors, numerous studies have reported an association between sepsis and a higher incidence of dementia.^42-44^ A 2022 meta-analysis of six studies reported sepsis survivors had a 1.62 times odds of all-cause dementia compared to patients without sepsis.^42^ Similarly, a cohort study in the United Kingdom^43^ reported adult sepsis survivors aged 65 and above had over double the risk of an incident dementia diagnosis compared to adults with no hospitalisation for infection. Fritze et al^44^ also demonstrated that German sepsis survivors had a higher risk of new dementia diagnoses for up to two years after sepsis compared to non-sepsis patients.

#### Potential Mechanisms for Cognitive Decline

There are many mechanisms through which sepsis may affect cognition, during both the initial hospitalisation and long-term recovery of survivors.^15,40,45^ Sepsis-induced endothelial dysfunction can result in disseminated intravascular coagulation as well as microcirculatory impairments, leading to ischemic brain lesions, cerebral infarcts, and stroke.^15,46^ In addition, dysregulation of the endothelium, blood brain barrier, and astrocytes in sepsis patients may allow entry of high and persistent levels of proinflammatory molecules, such as cytokines, leukocytes, and inflammasomes into the brain.^15,45^ This may dysregulate microglia function leading to further inflammation, and eventually cell death.^15,46^ Sepsis is also known to dysregulate metabolism, which can introduce neurotoxic compounds and molecules into the brain.^15,45^

In survivors of pediatric sepsis, approximately one in five had a sepsis-related MRI abnormality at ICU discharge, mostly related to white-matter signal abnormalities or ischemia, infarction, or thrombosis, and which were associated with new neurological disabilities.^47^ Similarly, Oh et al^48^ found the most commonly reported new disability in South Korean adult sepsis survivors were brain lesion disabilities. In elderly patients, these neuroinflammation and ischemic changes can exacerbate underlying, ‘silent’ pre-morbid conditions leading to further cognitive decline.^45^ A reduction in working memory capacity in sepsis survivors has also been linked to worse neurocognitive outcomes.^49^ These physiological changes may impair cognition,^15,45,46^ and could also cause depressive behaviours in sepsis survivors.^50^

### Mental and Emotional Health

While much of the research regarding sepsis and cognitive health is focused on cognitive decline, there is a growing body of literature investigating the mental and emotional health of survivors. In a recent qualitative study,^6^ survivors reported sepsis (i) impaired cognition causing fatigue, disorientation, and loss of previous skills, (ii) changed family and friend relationship dynamics, and (iii) caused anxiety about further illness, death, and their social relationships. Similarly, Apitzsch et al^51^ and Gallop et al^52^ identified changes in family and social interactions, poor information, traumatic sepsis hospitalisations, and the psychological impact on family/caregiver and survivor lives as key themes reported by Swedish, American, and British sepsis survivors.

#### Pediatric Sepsis Survivors

Compared to adult sepsis survivors, the mental health and emotional effects of sepsis in children are understudied. Two Dutch studies over a decade old found pediatric sepsis survivors reported depression or anxiety scores to be equal or better than those reported by reference populations or population norms.^53,54^ However, in survivors of pediatric sepsis, 25.7% and 15.3% of families reported persistently elevated distress and family dysfunction respectively over the 12-month follow-up period.^55^ Higher psychological distress in parents and caregivers was associated with worse quality of life outcomes in their sepsis-surviving children at one, three, and 12 months follow-up.^56^

#### Adult Sepsis Survivors

Adult patients have reported poorer psychological and mental health following sepsis.^37,57^ A prospective German cohort of adult ICU sepsis survivors found 44.2% and 40.9% self-reported a new onset of psychological impairment at one and three years after hospital discharge respectively.^37^ In comparison, a retrospective German cohort of administrative data using International Classification of Disease 10^th^ Edition, German modification (ICD-10-GM) codes reported 25.4% of patients without pre-existing mental health conditions reported a new diagnosis in the year after sepsis.^57^

##### Post Traumatic Stress Disorder (PTSD) in Sepsis Survivors

Sepsis is an acute and often traumatic medical event, and unsurprisingly post-traumatic stress disorder (PTSD) has been reported in survivors in both the immediate post-discharge period and years after.^57-59^ A 2024 study^59^ reported on three distinct clinical groups of PTSD-related presentations in sepsis survivors. Over a two year follow up period, 59% of survivors reported no or minimal PTSD symptoms, 15% recovered from PTSD symptoms, and 26% initially reported few symptoms at discharge but developed symptoms in the following two years.^59^ This highlights the importance of consistent and ongoing psychological screening and support for sepsis survivors.^59^ Female sex, ICU admission, and two or more traumatic memories of the ICU were associated with higher odds of developing PTSD symptoms.^57,59^

##### Depression and Suicide

Several studies have investigated the relationship between sepsis and depression,^60-64^ with varying results. In a cohort of 63 ICU patients in South Wales, 23 of which had sepsis, Battle et al^60^ reported sepsis patients had 6.8 times higher odds of depression. Two German studies found 27 of 224 (12.1%) sepsis survivors demonstrated depressive symptoms persisting for one year^64^ and 10.9% of 272 sepsis survivors with at least one sepsis sequalae had signs of depression six months post-discharge.^63^ A third retrospective cohort study from Germany reported 16.2% and 8.76% of ICU and non-ICU sepsis survivors respectively, had a new diagnosis of depression in the year following sepsis.^57^ Survivors who had been diagnosed with depression pre-sepsis were 18 times more likely to be diagnosed with depression again post-sepsis.^57^ Davydow et al^61^ found no association between sepsis and any subsequent depressive symptoms in a cohort of 439 US patients aged 50 and up, rather they found depression post-sepsis to be associated with depressive symptoms pre-sepsis. Similarly, in a cohort of 45,826 sepsis survivors in South Korea,^62^ 21% of patients had a diagnosis of depression before sepsis, with only an additional 2.4% receiving a new depression diagnosis within one year of surviving. A 2022 Australian study of 888 ICU patients, 282 with sepsis, found no difference in PTSD, anxiety, or depression between survivors of sepsis and survivors of other critical illnesses six months after ICU admission.^65^ They reported 20.5% of sepsis survivors had depression, compared to 17.3% of the critical illness survivors without sepsis.^65^

Each of these seven studies were set in different countries and used a diverse range of tools to identify depression, including ICD codes,^57,62^ the Hospital Anxiety and Depression Scale,^60,65^ the Major Depression Inventory,^64^ the Center for Epidemiologic Studies Depression Scale,^61^ and the Brief Symptom Inventory, BSI-18.^63^ This reflects the heterogeneity of the literature and may explain the variation in study outcomes linking depressive symptoms and sepsis. Overall, some common themes do emerge, with approximately 10–30% of survivors reporting depression symptoms,^57,62-65^ however more research is needed to confirm whether these symptoms are associated with sepsis,^60^ or largely due to other factors such as pre-sepsis mental health^57,61,62^ or ICU admission.^57^ Suicide has also been associated with sepsis. A study set in Denmark^66^ found sepsis infection to be associated with a 1.51 times higher risk of suicide than individuals without infection after adjusting for sex, age, calendar period, cohabitation status, socioeconomic status, and the Charlson Comorbidity Index (CCI). A 2022 South Korean study reported 1.5% of sepsis survivors died from suicide within a year of sepsis diagnosis.^67^

##### Mental and Emotional Health of Family and Friends

Sepsis does not only have the potential to impact the mental and emotional health of patients, but also of their family and friends.^52^ The relationships between sepsis survivors and their family and friends may change after discharge, potentially causing stress and anxiety for both patients and family.^6,52^ A study in 2013 by Rosendahl et al^68^ examined severe sepsis patient and spouse mental health after ICU care. Both patients and spouses reported high anxiety and depression scores, PTSD symptoms, and poor mental health related quality of life.^68^ Notably, PTSD symptoms in the patient influenced the reported quality of life in their spouse.^68^

### Changes in Functional Status and Daily Life

Many sepsis survivors report they can no longer perform activities they frequently enjoyed before sepsis, such as cooking, gardening, household chores, driving, shopping, social activities, reading, and working due to a lack of functional ability.^5,6,16,52^ The impact on daily life reported is often extremely diverse and can consist of both physical and mental effects.^5,35^ For example, survivors have reported fatigue, disorientation, memory, comprehension, mood, sensory, digestive, respiratory, cardiovascular, kidney, integumentary, and musculoskeletal concerns that did not exist prior to sepsis.^3,5,6,36^

#### Pediatric Sepsis Survivors

Current research into functional outcomes in pediatric sepsis survivors is limited. A study of Tanzanian pediatric survivors reported 4.3% had a decline in functional status at 28 days as measured by a change of three or more in the functional status scale.^69^ However, in the same study 13.8% of children's parents/guardians reported they perceived their child was still not back to previous health at 28 days.^69^ In comparison, another study set across 18 countries^70^ reported 34% of pediatric sepsis survivors had a decline in functional status at 28 days from hospital discharge.

#### Adult Sepsis Survivors

Studies report between half and three quarters of adult sepsis survivors suffer from a functional disability, fatigue, or an increase in comorbidities following sepsis.^48,63,71,72^ Within one year of hospital discharge, 52.2% of a cohort of 209 sepsis patients had a severe disability or died, rising over 2.5 times from the 18.1% who had a severe disability before their sepsis admission.^72^ In contrast, a 2022 study in Australia reported no difference in the risk of new disabilities at three or six months after adjusting for covariates between critically ill patients with and without sepsis.^65^ At three months post discharge sepsis survivor muscle strength is also lower than expected with survivors showing reduced capacity for physical activity compared to healthy controls.^73^ Furthermore, after three years 58.5% of ICU sepsis survivors report muscular weakness.^37^ These issues can extend to dysphagia, or difficulty swallowing, which has been reported to affect between one in twelve and one in three sepsis survivors on hospital discharge^74,75^ and one in seven ICU sepsis survivors after three years.^37^

Many sepsis survivors lose their independence after sepsis, leading to feelings of frustration, helplessness, and embarrassment.^52^ Survivors often rely upon family members to support their new care needs.^6,51,52^ Yende et al^76^ reported that in a previously independent population many sepsis patients who survived to six months post-sepsis had not returned to independent living. Similarly, Ehlenbach et al^77^ found almost three quarters of survivors discharged to a US skilled nursing facility after sepsis were maximally or totally dependent for activities of daily living. A German cohort reported the probability of remaining functionally dependent, regaining independence, or dying within 3 years of hospital discharge was 20.5%, 32.3%, and 47.2% in 753 ICU sepsis survivors respectively.^37^ At three years, 46.4% and 37% of ICU sepsis survivors reported walking difficulties and fatigue.^37^ These functional issues may be more apparent in older survivor populations. A 2024 narrative review found older sepsis survivors often suffered from long term functional declines such as muscle weakness, fatigue, cognitive decline, sleep disturbances, emotional distress, and swallowing issues.^18^

### Quality of Life Impacts

There is a growing appreciation for the importance of health-related quality of life (HRQoL) as an outcome for evaluating both sepsis survivorship and critical care.^78,79^ A systematic review of 48 studies demonstrated that HRQoL was worse for survivors of critical illness compared to population norms.^78^

#### Pediatric Sepsis Survivors

There are comparatively few studies investigating the HRQoL of pediatric sepsis survivors compared to adults. A large US cohort study of 389 children across 12 PICUs reported 35% of surviving patients did not return to their baseline HRQoL by 12 months using the Stein-Jessop Functional Status II-R Short Form (FSII-R) or the Pediatric Quality of Life Inventory 4.0 Generic Core Scales (PedsQL).^80-82^ Another US study published in 2019 reported that 23.8% of 790 pediatric sepsis patients did not return to their baseline HRQoL by the follow-up timepoint (two to 12 weeks post-discharge) as measured by the PedsQL.^83^ The same study reported that 34.9% of pediatric sepsis patients returned to baseline and 41.5% reported a HRQoL above baseline.^83^ A Dutch study, published in 2009, contradicted these results, reporting HRQoL in 50 pediatric survivors aged 8–18 years as comparable to the population norm using KIDSCREEN-52.^53^

The contradicting HRQoL results between these studies could be attributed to a range of factors including the different HRQoL tools used, or the differences in the study populations and the services received. Both the studies reporting a change in HRQoL were conducted in the US and used PedsQL or FSII-R, whereas the study reporting no change was set in the Netherlands, used KIDSCREEN-52 and published a decade before the US studies. Some of the risk factors associated with worse HRQoL in pediatric sepsis survivors included single parenthood of children with developmental disabilities,^82^ a longer hospital stay^,^^81^ neurological injury or abnormality,^81^ treatment for intracranial pressure,^81^ medical device use at one month,^81^ severity of organ dysfunction,^84^ decreased functional status score,^85^ and older age.^81,84^

#### Adult Sepsis Survivors

Adult survivors of sepsis consistently report worse HRQoL compared to the population norm,^86,87^ and their own baseline.^88^ However, when compared to non-septic critical care survivors, sepsis survivors often do not report a lower HRQoL.^89,90^ A systematic review found 12 of 16 studies comparing HRQoL in sepsis ICU survivors to age/gender/general matched populations reported significant declines in physical and social HRQoL domains.^91^ Whereas, four studies comparing sepsis survivors to other critically ill ICU survivors, reported similar HRQoL scores.^91^ This suggests that the impact of sepsis on HRQoL may be similar to that of other critical illnesses. Risk factors identified for poorer HRQoL in adult survivors included: older age,^87,89^ female sex,^87,89^ poor physical health^92^ (such as extended ongoing illness,^93,94^ severe frailty,^88^ neurological organ dysfunction,^87^ and previous comorbidity^87^), hospitalisation factors (such as increased hospital length of stay,^87^ and longer mechanical ventilation^89^), spirituality,^93^ poor mental health^92^ (such as higher stress^93^), inadequate social support,^92^ and neutrophil-lymphocyte biomarkers.^92^

However, HRQoL assessment forms may not accurately measure the experience of sepsis survivors. While traditional HRQoL assessment forms, such as SF-36, have shown validity and reliability when tested in sepsis survivors,^95^ a qualitative study interviewing sepsis survivors reported many important concerns were not adequately captured by these traditional survey instruments.^79^ Survivors reported 11 domains as critically important, seven of which were not included in traditional HRQoL tools: return to normal living, ability to walk, cognitive impairment, self-perception, control over one's life, family support, and delivery of healthcare.^79^

## Discussion

This review comprehensively summarises the wide-reaching quality of life and cognitive, mental, and functional health impacts sepsis survivors face at all ages, as well as the considerable burden these morbidities place on patients and families. Two key areas for targeted further research were identified.

The first key area is non-neonatal pediatric sepsis survivors. Our findings found little research on the cognitive, mental health, and functional impacts of sepsis in non-neonatal pediatric survivors. While there has been extensive research on neurodevelopmental impairments in preterm neonates, this is only one aspect of the morbidity burden in survivors of pediatric and neonatal sepsis. In comparison, far more studies investigated adult survivor populations. This is concerning, as there is evidence that brain damage at a young age cumulatively affects a child's neurodevelopment, with more cognitive and mental health impacts being revealed over time (known as the “growing into deficits” hypothesis).^33,54^ Further robust research aimed at elucidating the mental, cognitive, and functional health burden on survivors of childhood sepsis is needed to provide a more complete understanding of sepsis survivorship in children and throughout development into adulthood.

The second key area is the mental health morbidity burden in survivors. We identified the mental health impact of sepsis as a research gap across all ages. In particular, future research should investigate the burden of anxiety disorders and symptoms as a recent qualitative study found anxiety was a key theme reported by survivors.^6^ Furthermore, a systematic review of 27 studies investigating anxiety and post intensive care syndrome (PICS) reported between 32% to 40% of ICU survivors had anxiety symptoms through their first-year post discharge.^96^ As, on average, up to a third of intensive care unit patients have sepsis,^13^ it is likely there are similarities in mental health impacts. Investigating the impact of sepsis on anxiety and other aspects of mental health in survivors of all ages is needed to better understand the extent of the mental health morbidity burden post-sepsis. In addition, there is recent evidence to indicate that traditional quality of life instruments do not accurately capture all dimensions relevant to the quality of life of sepsis survivors.^79^ Further robust research should be performed to investigate better fitting quality of life instruments, able to accurately reflect sepsis survivor experiences.

Regular post-discharge cognitive and psychological screening should be an integral part of care to ensure survivors are supported emotionally and cognitively during their recovery.^15,51,97^ This support should also be offered to family members, as studies have reported poorer mental and emotional health in survivor families.^55,56,68^ However, survivors have previously reported inadequate sepsis-specific healthcare support.^5,79,98^ An international survey reported almost a third (29.3%) of 1731 adult survivors across 41 countries were not provided with any social services support after leaving hospital.^5^ Furthermore, adult survivors in the United States,^52^ United Kingdom,^52^ Germany,^79^ and Sweden^51^ reported a critical lack of information provided on post-sepsis recovery and difficulty finding or accessing health services specific to their needs. While physical rehabilitation is starting to be provided consistently, cognitive, mental, and functional health concerns are still often not addressed.^5,98^ Gawlytta et al (2022) investigated a program using internet cognitive behavioural therapy to improve PTSD symptoms and health-related quality of life in adult survivors, however, did not find a difference between the intervention and control groups.^99^

Many current studies focus on rehabilitation interventions for adult sepsis patients that are implemented during the original hospitalisation and designed to improve functional outcomes, but these have shown varied success.^100-107^ A systematic review of two randomised controlled trials investigated assisted exercise and neuromuscular stimulation during ICU stay, finding some evidence of improved quality of life but no improvement in length of stay, muscle strength, or ICU mortality.^108^ Other studies reported improved long-term survival with in-patient rehabilitation and physiotherapy-led programs.^109-111^ The Sepsis Transition and Recovery (STAR) program, set in the US, had a nurse deliver discharge and infection-specific education to sepsis patients, and provide post-discharge care over the telephone.^112^ When evaluated the STAR program demonstrated improved 30-day mortality, 30-day readmission, and 12-month mortality and readmission composite outcomes.^112,113^ A German program, the SMOOTH trial, improved discharge management, delivered education about sepsis sequalae to clinicians and patients, and provided monthly telephone calls.^114^ The SMOOTH trial did not demonstrate any improvement in mental health related quality of life or change primary clinician care but was potentially protective against PTSD symptoms.^114,115^

Only two studies reported interventions aimed specifically at pediatric sepsis survivors.^116,117^ However, neither report health outcomes, with one a protocol for the implementation of a government program for family and parent support in Australia,^117^ and the other a description of the successful implementation of a follow up program for pediatric sepsis survivors in the US.^116^ Little to no research investigates in-hospital or post-hospital rehabilitation programs for pediatric and neonatal sepsis patients.^118^ Pediatric survivors represent a substantial part of the sepsis population burden and suffer from many sequalae as described earlier in this review. Therefore, interventions for post-sepsis rehabilitation and recovery in infants and children who survive sepsis is a critical area for future research.

In conclusion, survivors of sepsis often suffer from a diverse range of ongoing cognitive, mental, and functional health concerns and poorer quality of life. Key areas to target future research are the cognitive and functional morbidity burden in survivors of childhood sepsis, and the mental health burden in survivors of all ages. A better understanding of the burden of morbidities in survivors could assist in improving carer, patient, and clinician vigilance in identifying and seeking treatment for sepsis related sequalae. Such new evidence could inform interventions aimed at improving survivor quality of life and cognitive, mental, and functional health, potentially leading to improved survivor rehabilitation.

## Supplemental Material

sj-pdf-1-jic-10.1177_08850666251340631 - Supplemental material for Cognitive Health and Quality of Life After Surviving Sepsis: A Narrative Review Supplemental material, sj-pdf-1-jic-10.1177_08850666251340631 for Cognitive Health and Quality of Life After Surviving Sepsis: A Narrative Review by Khalia Ackermann, MPH, Nanda Aryal, PhD, Johanna Westbrook, PhD, and Ling Li, PhD in Journal of Intensive Care Medicine
