# Supplementary material for: Cognitive Health and Quality of Life After Surviving Sepsis: A Narrative Review
Source: J Intensive Care Med. 2025 May 16;41(10):936–46. doi: 10.1177/08850666251340631 (PMC13538917; doi:10.1177/08850666251340631)
Supplement: Supplementary file 1 [file sj-pdf-1-jic-10.1177_08850666251340631.pdf]

Appendix A – MEDLINE search strategies

Cognitive impairment and decline

1	sepsis/ or neonatal sepsis/ or shock, septic/ or systemic inflammatory response syndrome/
2	("systemic inflammatory response syndrome" or sepsis or SIRS or septi* or "septic shock").mp. /freq=3
3	1 or 2
4	Survivors/
5	(“post sepsis syndrome” or surviv*).mp.
6	4 or 5
7	Child Development/ or Alzheimer Disease/ or Mental Disorders/ or Dementia/ or Cognitive Dysfunction/ or Cognition Disorders/ or Intellectual Disability/ or Cognition/ or Brain/ or Brain Injuries/
8	("neurodevelopmental impairment" or cogniti* or development or dementia or alzheimer or "mental disorder" or disab* or brain or neuro* or cerebral).mp. /freq=3
9	7 or 8
10	exp animals/ not humans.sh.
11	(mouse or rat or rats or mice or monkey or ape or dog or cat or murine or swine or "in vitro").mp. /freq=3
12	10 or 11
13	3 and 6 and 9
14	13 not 12

Mental and emotional health

1	sepsis/ or neonatal sepsis/ or shock, septic/ or systemic inflammatory response syndrome/
2	("systemic inflammatory response syndrome" or sepsis or SIRS or septi* or "septic shock").mp. /freq=3
3	1 or 2
4	Survivors/
5	(“post sepsis syndrome” or surviv*).mp.
6	4 or 5
7	Mental Health/ or Depression/ or Anxiety Disorders/ or Anxiety/ or Stress Disorders, Post-Traumatic/
8	("mental health" or anxiety or depressi* or stress or "post traumatic stress disorder" or PTSD).mp. /freq=3
9	7 or 8
10	exp animals/ not humans.sh.
11	(mouse or rat or rats or mice or monkey or ape or dog or cat or murine or swine or "in vitro").mp. /freq=3
12	10 or 11
13	3 and 6 and 9
14	13 not 12

Quality of life impacts

1	sepsis/ or neonatal sepsis/ or shock, septic/ or systemic inflammatory response syndrome/
2	("systemic inflammatory response syndrome" or sepsis or SIRS or septi* or "septic shock").mp. /freq=3
3	1 or 2
4	"Quality of Life"/ or Quality-Adjusted Life Years/
5	("Quality of life" or "health related quality of life" or "quality adjusted life year*" or QALY* or QoL or HRQoL).mp. /freq=3
6	4 or 5
7	3 and 6

Changes in functional status and daily life

1	sepsis/ or neonatal sepsis/ or shock, septic/ or systemic inflammatory response syndrome/
2	("systemic inflammatory response syndrome" or sepsis or SIRS or septi* or "septic shock").mp. /freq=3
3	1 or 2
4	Survivors/
5	(“post sepsis syndrome” or surviv*).mp.
6	4 or 5
7	"Activities of Daily Living"/ or Functional Status/ or Fatigue/ or Muscle Fatigue/ or Mental Fatigue/ or Fatigue Syndrome, Chronic/ or Muscle Weakness/ or Cognitive Dysfunction/ or Independent Living/
8	(fatigue or "brain fog" or "functional status" or "activities of daily living" or "muscle weakness").mp. /freq=3
9	7 or 8
10	exp animals/ not humans.sh.
11	(mouse or rat or rats or mice or monkey or ape or dog or cat or murine or swine or "in vitro").mp. /freq=3
12	10 or 11
13	3 and 6 and 9
14	13 not 12

Appendix B – Included studies

First author (publication year)	Title	Number of participants	Population	Study design^	Review outcome category
Al Khalaf (2015) [72]	Determinants of functional status among survivors of severe sepsis and septic shock: One-year follow-up	209	Adult	Cohort	Functional status and daily life
Als (2013) [29]	Neuropsychologic function three to six months following admission to the PICU with meningoencephalitis, sepsis, and other disorders: a prospective study of school-aged children	188	Pediatric	Case-control	Cognitive impairment and decline
Alshaikh (2013) [25]	Neurodevelopmental outcomes of very low birth weight infants with neonatal sepsis: systematic review and meta-analysis	21,629 (17 studies)	Pediatric	Systematic review and meta-analysis	Cognitive impairment and decline
Annane (2015) [15]	Cognitive decline after sepsis	N/A	Both	Narrative Review	Cognitive impairment and decline
Apitzsch (2021) [51]	The physical and mental impact of surviving sepsis – a qualitative study of experiences and perceptions among a Swedish sample	8	Adult	Interview	Mental and emotional health<br><br>Functional status and daily life
Atterton (2020) [41]	Sepsis Associated Delirium	N/A	Adult[#]	Narrative Review	Cognitive impairment and decline
Bakhuizen (2014) [26]	Meta-analysis shows that infants who have suffered neonatal sepsis face an increased risk of mortality and severe complications	28,392 (12 studies)	Pediatric	Systematic review and meta-analysis	Cognitive impairment and decline
Barichello (2022) [46]	Postmortem Evidence of Brain Inflammatory Markers and Injury in Septic Patients: A Systematic Review	422 (104 studies)	Both	Systematic review	Cognitive impairment and decline
Battle (2015) [60]	Depression following critical illness: Analysis of incidence and risk factors	63	Adult	Survey	Mental and emotional health
Becker (2022) [47]	Sepsis-Related Brain MRI Abnormalities Are Associated With Mortality and Poor Neurological Outcome in Pediatric Sepsis	140	Pediatric	Cohort	Cognitive impairment and decline
Boede (2021) [64]	Trajectories of depression in sepsis survivors: an observational cohort study	224	Adult	Cohort	Mental and emotional health
Borges (2015) [73]	Physical activity, muscle strength, and exercise capacity 3 months after severe sepsis and septic shock	122	Adult	Cohort	Functional status and daily life

Bronner (2009) [53]	An explorative study on quality of life and psychological and cognitive function in pediatric survivors of septic shock	50	Pediatric	Cohort	Mental and emotional health<br><br>Quality of life impacts
Cai (2019) [24]	Short- and Long-Term Neurodevelopmental Outcomes of Very Preterm Infants with Neonatal Sepsis: A Systematic Review and Meta-Analysis	19,582 (24 studies)	Pediatric	Systematic review and meta-analysis	Cognitive impairment and decline
Calsavara (2018) [34]	Post-sepsis cognitive impairment and associated risk factors: A systematic review	74,313,495 (16 studies)	Adult	Systematic review and meta-analysis	Cognitive impairment and decline
Calsavara (2021) [58]	Prevalence and risk factors for post-traumatic stress, anxiety, and depression in sepsis survivors after ICU discharge	33	Adult	Interview	Mental and emotional health
Chung (2020) [17]	Sepsis-Associated Encephalopathy: From Delirium to Dementia?	N/A	Adult[#]	Narrative review	Cognitive impairment and decline
Davydow (2013) [61]	Symptoms of Depression in Survivors of Severe Sepsis: A Prospective Cohort Study of Older Americans	439	Adult	Cohort	Mental and emotional health
Dong (2023) [88]	Quality of life and mortality in older adults with sepsis after one-year follow up: A prospective cohort study demonstrating the significant impact of frailty	211	Adult	Cohort	Quality of life impacts
Ehlenbach (2018) [77]	Sepsis Survivors Admitted to Skilled Nursing Facilities: Cognitive Impairment, Activities of Daily Living Dependence, and Survival	135,370	Adult	Cohort	Functional status and daily life
Farris (2013) [70]	Functional Outcomes in Pediatric Severe Sepsis: Further Analysis of the Researching Severe Sepsis and Organ Dysfunction in Children: A Global Perspective Trial	384	Pediatric	Cohort	Functional status and daily life
Fleischmann-Struzek (2021) [36]	Epidemiology and Costs of Postsepsis Morbidity, Nursing Care Dependency, and Mortality in Germany, 2013 to 2017	159,684	Adult	Cohort	Cognitive impairment and decline<br>Functional status and daily life
Fleischmann-Struzek (2024) [37]	Functional dependence following intensive care unit-treated sepsis: three-year follow-up results from the prospective Mid-German Sepsis Cohort (MSC)	753	Adult	Cohort	Cognitive impairment and decline<br>Mental and emotional health<br>Functional status and daily life

Fritze (2021) [44]	Time course of dementia following sepsis in German health claims data	161,567	Adult	Cohort	Cognitive impairment and decline
Gallop (2015) [52]	A qualitative investigation of patients' and caregivers' experiences of severe sepsis*	39	Adult	Interview	Mental and emotional health<br><br>Functional status and daily life
Gardner (2019) [94]	The Development of Chronic Critical Illness Determines Physical Function, Quality of Life, and Long-Term Survival Among Early Survivors of Sepsis in Surgical ICUs	173	Adult	Cohort	Quality of life impacts
Gerth (2019) [78]	Changes in health-related quality of life after discharge from an intensive care unit: a systematic review	18,011	Adult	Systematic review	Quality of life impacts
Heyland (2000) [95]	Long-term health-related quality of life in survivors of sepsis. Short Form 36: a valid and reliable measure of health-related quality of life	30	Adult	Interview	Quality of life impacts
Higgins (2019) [86]	Quality of Life and 1-Year Survival in Patients With Early Septic Shock: Long-Term Follow-Up of the Australasian Resuscitation in Sepsis Evaluation Trial	1,591	Adult	Randomised control trial	Quality of life impacts
Hodgson (2022) [65]	Comparison of 6-month outcomes of sepsis versus non-sepsis critically ill patients receiving mechanical ventilation	888	Adult	Cohort	Mental and emotional health<br><br>Functional status and daily life
Hongo (2023) [75]	Frequency, associated factors, and associated outcomes of dysphagia following sepsis	101	Adult	Cohort	Functional status and daily life
Huang (2019) [5]	Life after sepsis: an international survey of survivors to understand the post-sepsis syndrome	1,731	Adult[#]	Survey	Functional status and daily life
Humberg (2022) [28]	Recurrent Late-Onset Sepsis in Extremely Low Birth Weight Infants Is Associated with Motor Deficits in Early School Age	1,343	Pediatric	Cohort	Cognitive impairment and decline
Ibarz (2024) [18]	The critically ill older patient with sepsis: a narrative review	N/A	Adult	Narrative review	Functional status and daily life
Iwashyna (2010) [35]	Long-term Cognitive Impairment and Functional Disability Among Survivors of Severe Sepsis	5,033	Adult	Cohort	Cognitive impairment and decline Functional status and daily life

Iwashyna (2012) [71]	Population burden of long-term survivorship after severe sepsis in older Americans	637,867	Adult	Cohort	Functional status and daily life
Kachmar (2018) [33]	A Systematic Review of Risk Factors Associated With Cognitive Impairment After Pediatric Critical Illness	1,912 (12 studies)	Pediatric	Systematic review	Cognitive impairment and decline
Kattlun (2024) [49]	Persistent neurocognitive deficits in cognitively impaired survivors of sepsis are explained by reductions in working memory capacity	73	Adult	Cohort	Cognitive impairment and decline
Kaur (2016) [30]	Neurodevelopmental and Behavioral Outcomes in Children With Sepsis-Associated Encephalopathy Admitted to Pediatric Intensive Care Unit: A Prospective Case Control Study	100	Pediatric	Case Control	Cognitive impairment and decline
Killien (2019) [83]	Health-Related Quality of Life Among Survivors of Pediatric Sepsis	790	Pediatric	Cohort	Quality of life impacts
Killien (2022) [56]	Association of Illness Severity With Family Outcomes Following Pediatric Septic Shock	276	Pediatric	Cohort	Mental and emotional health
König (2019) [79]	What matters most to sepsis survivors: a qualitative analysis to identify specific health-related quality of life domains	15	Adult	Interviews	Quality of life impacts
Kurematsu (2023) [93]	Quality of Life of Sepsis Survivors	81	Adult	Cohort	Quality of life impacts
Kurul (2023) [27]	Inflammation, sepsis severity and neurodevelopmental outcomes of late-onset sepsis in preterm neonates	410	Pediatric	Cohort	Cognitive impairment and decline
Lau-Braunhut (2021) [69]	Functional Outcomes and Morbidity in Pediatric Sepsis Survivors: A Tanzanian Experience	1,359	Pediatric	Cohort	Functional status and daily life
Lei (2022) [42]	Risk of Dementia or Cognitive Impairment in Sepsis Survivals: A Systematic Review and Meta-Analysis	891,562 (8 studies)	Adult	Systematic review and meta-analysis	Cognitive impairment and decline
Lund-Sørensen (2016) [66]	A Nationwide Cohort Study of the Association Between Hospitalization With Infection and Risk of Death by Suicide	7,221,578	Adult	Cohort	Mental and emotional health
Manabe (2022) [45]	Cerebral dysfunctions caused by sepsis during ageing	N/A	Adult	Narrative review	Cognitive impairment and decline
Mazeraud (2020) [40]	Septic-Associated Encephalopathy: a Comprehensive Review	N/A	Adult	Narrative review	Cognitive impairment and decline
Meert (2020) [81]	Trajectories and Risk Factors for Altered Physical and Psychosocial Health-Related Quality of Life After Pediatric Community-Acquired Septic Shock	204	Pediatric	Cohort	Quality of life impacts

Meert (2021) [82]	Health-Related Quality of Life After Community-Acquired Septic Shock in Children With Preexisting Severe Developmental Disabilities	137	Pediatric	Cohort	Quality of life impacts
Mostel (2019) [3]	Post-sepsis syndrome – an evolving entity that afflicts survivors of sepsis	N/A	Adult	Narrative review	Functional status and daily life
Murphy (2020) [55]	Longitudinal Trajectories of Caregiver Distress and Family Functioning After Community-Acquired Pediatric Septic Shock	260	Pediatric	Cohort	Mental and emotional health
Muzambi (2021) [43]	Assessment of common infections and incident dementia using UK primary and secondary care data: a historical cohort study	989,800	Adult	Cohort	Cognitive impairment and decline
Nannan Panday (2019) [87]	Health related quality of life in sepsis survivors from the Prehospital Antibiotics Against Sepsis (PHANTASi) trial	880	Adult	Cohort	Quality of life impacts
Nanayakkara (2017) [91]	Long-term health related quality of life in patients with sepsis after intensive care stay: A systematic review	5,333 (16 studies)	Adult	Systematic review	Quality of life impacts
Oh (2021) [62]	Depression and Long-Term Survival Among South Korean Sepsis Survivors: A Nationwide Cohort Study From 2011 to 2014	45,826	Adult	Cohort	Mental and emotional health
Oh (2021) [48]	Quality of life after sepsis and its association with mortality among sepsis survivors in South Korea: A population level cohort study	119,660	Adult	Cohort	Cognitive impairment and decline<br><br>Functional status and daily life
Oh (2022) [67]	Prevalence and risk factors for suicide in patients with sepsis: nationwide cohort study in South Korea	251,837	Adult	Cohort	Mental and emotional health
Pongsuwun (2024) [92]	Factor Impacting Quality of Life Among Sepsis Survivors During and After Hospitalization: A Systematic Review of Current Empirical Evidence	122,638 (8 studies)	Adult	Systematic Review	Quality of life impacts
Rosendahl (2013) [68]	Physical and mental health in patients and spouses after intensive care of severe sepsis: a dyadic perspective on long-term sequelae testing the Actor-Partner Interdependence Model	110	Adult	Cohort	Mental and emotional health
Sankar (2021) [32]	Functional Outcomes at 1 Year After PICU Discharge in Critically Ill Children With Severe Sepsis	121	Pediatric	Cohort	Cognitive impairment and decline

Sasegbon (2018) [74]	Examining the relationship between sepsis and oropharyngeal dysphagia in hospitalised elderly patients: a retrospective cohort study	301	Adult	Cohort	Functional status and daily life
Savioli (2018) [31]	Suspected or known neonatal sepsis and neurodevelopmental delay by 5 years	65,938	Pediatric	Cohort	Cognitive impairment and decline
Schade Skov (2024) [6]	How do sepsis survivors experience life after sepsis? A Danish qualitative study exploring factors of importance	16	Adult	Interview	Mental and emotional health Functional status and daily life
Schmidt (2024) [59]	Trajectories of post-traumatic stress in sepsis survivors two years after ICU discharge: a secondary analysis of a randomized controlled trial	175	Adult	Cohort	Mental and emotional health
Seidel (2020) [39]	Accelerated brain ageing in sepsis survivors with cognitive long-term impairment	64	Adult[#]	Cohort	Cognitive impairment and decline
Sell (2025) [57]	Mental health in the first year after ICU-treated sepsis: Analysis of administrative diagnoses in German health claims data	21,980	Adult	Cohort	Mental and emotional health
Sewell (2021) [19]	Association of Infection in Neonates and Long-Term Neurodevelopmental Outcome	N/A	Pediatric	Narrative review	Cognitive impairment and decline
Stallmach (2022) [63]	Comparison of fatigue, cognitive dysfunction and psychological disorders in post-COVID patients and patients after sepsis: is there a specific constellation?	272	Adult	Cohort	Mental and emotional health<br><br>Functional status and daily life
Su (2018) [89]	Long-term quality of life after sepsis and predictors of quality of life in survivors with sepsis	612	Adult	Case control	Quality of life impacts
Thompson (2018) [90]	Health-related outcomes of critically ill patients with and without sepsis	1,612	Adult	Cohort	Quality of life impacts
Van der Slikke (2020) [16]	Exploring the pathophysiology of post-sepsis syndrome to identify therapeutic opportunities	N/A	Adult	Narrative review	Functional status and daily life
Vermunt (2008) [54]	Behavioural, emotional, and post-traumatic stress problems in children and adolescents, long term after septic shock caused by Neisseria meningitidis	1,690	Pediatric	Survey/Interview	Mental and emotional health
Wang (2021) [38]	Trajectory of Cognitive Decline After Sepsis	21,823	Adult	Cohort	Cognitive impairment and decline
Wang (2023) [50]	Recent advances in the study of sepsis-induced depression	N/A	Adult	Narrative review	Mental and emotional health

Workman (2023) [85]	Change in Functional Status During Hospital Admission and Long-Term Health-Related Quality of Life Among Pediatric Septic Shock Survivors	224	Pediatric	Cohort	Quality of life impacts
Yende (2016) [76]	Long-Term Quality of Life Among Survivors of Severe Sepsis: Analyses of Two International Trials	2,130	Adult	Cohort	Functional status and daily life
Zimmerman (2020) [80]	Trajectory of Mortality and Health-Related Quality of Life Morbidity Following Community-Acquired Pediatric Septic Shock	389	Pediatric	Cohort	Quality of life impacts
Zimmerman (2020) [84]	Critical Illness Factors Associated With Long-Term Mortality and Health-Related Quality of Life Morbidity Following Community-Acquired Pediatric Septic Shock	389	Pediatric	Cohort	Quality of life impacts

^If the study did not clearly state study design, we used the study flow chart from Ackermann et al. (2022) to characterise studies (all cohort studies were simply labelled “cohort study”). Reviews were categorized as systematic, scoping, or narrative as appropriate.

[#]If the participant population is not specified or easily inferred from study context, it was assumed to be adult.

Ackermann, K., Baker, J., Green, M., Fullick, M., Varinli, H., Westbrook, J., & Li, L. (2022). Computerized Clinical Decision Support Systems for the Early Detection of Sepsis Among Adult Inpatients: Scoping Review. *Journal of medical Internet research*, *24*(2), e31083. https://doi.org/10.2196/31083
